# Identification of prognostic alternative splicing events related to the immune microenvironment of hepatocellular carcinoma

**DOI:** 10.1186/s10020-021-00294-3

**Published:** 2021-04-08

**Authors:** Shanshan Yu, Luya Cai, Chuan Liu, Ruihong Gu, Lingyi Cai, Leying Zhuo

**Affiliations:** 1 Department of Chemoradiation Oncology, The First Affiliated Hospital of Wenzhou Medical University, Wenzhou, Zhejiang People’s Republic of China; 2 Department of Obstetrics and Gynecology, The First Affiliated Hospital of Wenzhou Medical University, Wenzhou, Zhejiang People’s Republic of China; 3 Department of Medical Oncology, The First Hospital of China Medical University, Shenyang, Liaoning People’s Republic of China; 4 Department of Microbiology and Immunology, Institute of Molecular Virology and Immunology, Institute of Tropical Medicine, School of Basic Medical Sciences, Wenzhou Medical University, Wenzhou, Zhejiang People’s Republic of China; 5 Department of Hematology, The Second Affiliated Hospital of Wenzhou Medical University, Wenzhou, Zhejiang People’s Republic of China; 6 Department of Intensive Care Unit, The First Affiliated Hospital of Wenzhou Medical University, Southern White Elephant Town, Ouhai, Wenzhou, Zhejiang 325000 People’s Republic of China

**Keywords:** Hepatocellular carcinoma, Alternative splicing, Prognosis, Carcinogenesis, Immune microenvironment

## Abstract

**Background:**

Hepatocellular carcinoma (HCC) is one of the most common malignant tumors in the world, and its 5-year survival rate is less than 20%, despite various treatments being available. Increasing evidence indicates that alternative splicing (AS) plays a nonnegligible role in the formation and development of the tumor microenvironment (TME). However, the comprehensive analysis of the impact on prognostic AS events on immune-related perspectives in HCC is lacking but urgently needed.

**Methods:**

The transcriptional data and clinical information of HCC patients were downloaded from TCGA (The Cancer Genome Atlas) database for calculating immune and stromal scores by ESTIMATE algorithm. We then divided patients into high/low score groups and explored their prognostic significance using Kaplan–Meier curves. Based on stromal and immune scores, differentially expressed AS events (DEASs) were screened and evaluated with functional enrichment analysis. Additionally, a risk score model was established by applying univariate and multivariate Cox regression analyses. Finally, gene set variation analysis (GSVA) was adopted to explore differences in biological behaviors between the high- and low-risk subgroups.

**Results:**

A total of 370 HCC patients with complete and qualified corresponding data were included in the subsequent analysis. According to the results of ESTIMATE analysis, we observed that the high immune/stromal score group had a longer survival probability, which was significantly correlated with prognosis in HCC patients. In addition, 467 stromal/immune score-related DEASs were identified, and enrichment analysis revealed that DEASs were significantly enriched in pathways related to HCC tumorigenesis and the immune microenvironment. More importantly, the final prognostic signature containing 16 DEASs showed powerful predictive ability. Finally, GSVA demonstrated that activation of carcinogenic pathways and immune-related pathways in the high-risk group may lead to poor prognosis.

**Conclusions:**

Collectively, these outcomes revealed prognostic AS events related to carcinogenesis and the immune microenvironment, which may yield new directions for HCC immunotherapy.

**Supplementary Information:**

The online version contains supplementary material available at 10.1186/s10020-021-00294-3.

## Background

Hepatocellular carcinoma (HCC) is the fourth most common cause of cancer-related deaths, ranking sixth in new cancer cases worldwide (Villanueva and Longo 2019). According to GLOBOCAN statistics for 2018, the global number of patients with HCC was 854,000, while the number of deaths was 781,000 (Bray et al. 2018). The morbidity and mortality are so close that the five-year survival rate is only 18%, making it the second deadliest tumor after pancreatic cancer (Jemal et al. 2017). At present, treatment of HCC remains surgery-based comprehensive treatment, but most patients are usually diagnosed in a late stage due to a lack of typical clinical manifestations in early HCC (Heimbach et al. 2018; Bruix and Sherman 2011). Even more disappointing is that although there are many treatment options for advanced HCC, such as radiochemotherapy, interventional therapy, ablation therapy, targeted therapy and antiangiogenic treatment, they only provide moderate benefits for HCC patients (Qin et al. 2013; Kudo et al. 2018). Therefore, patients with HCC are in urgent need of newer and more effective treatments.

The emergence of immunotherapy has revolutionized the traditional mode of diagnosis and treatment, bringing hope to advanced HCC patients. Immune checkpoint inhibitors based on PD-1/PD-L1 significantly extend patient survival, and in particular, PD-1/PD-L1 inhibitors have an amazing synergistic effect when combined with antiangiogenic drugs (Zhu et al. 2018; Yau et al. 2020). However, only a small number of patients receiving immunotherapy treatment respond to the treatment, which is limited by the immunosuppressive microenvironment of HCC (Zhang et al. 2020). HCC is a typical inflammation-related malignancy whose microenvironment contains a large number of macrophages and a series of innate and adaptive immune cells, forming a complex immune-tolerant microenvironment (Kurebayashi et al. 2018; Nishida and Kudo 2017). Understanding the mechanism of the formation and transformation of each cell component in the HCC microenvironment is essential for identifying new diagnostic, prognostic and therapeutic targets.

With the rapid development of next-generation sequencing technology, it has become a trend to use big data based on tumor genomics to mine and analyze the internal factors that affect the formation and development of the tumor microenvironment (Nacev et al. et al. 2019; Francisco Sanchez-Vega et al. 2018; Li et al. 2019). Alternative splicing (AS) refers to the process of splicing a single RNA precursor in different ways to produce different structural and functional mRNA and protein variants and may be one of the most widely used mechanisms to explain proteome diversity and cellular complexity (Climente-González et al. 2017). More intriguingly, approximately 95% of human genes undergo some level of AS in physiological processes according to genome-wide studies, in which some abnormal AS events may be considered potential drivers of tumorigenesis (Climente-González et al. 2017; Xiong et al. 2014). Increasing evidence shows that AS not only has a significant relationship with tumor occurrence and development, invasion and metastasis, and treatment resistance but also plays an important role in the formation of the immune microenvironment (Wan et al. 2019; Oltean and Bates 2013; Yao et al. 2016; Qi et al. 2020). In other words, in addition to affecting the infiltration of immune cells, changes in AS can also regulate tumor-related immunocytolytic activity (Li et al. 2019). However, to the best of our knowledge, although there are also some studies based on AS events in HCC (Yang et al. 2019a; Xiong et al. 2020; Lee et al. 2020), there is a scarcity of studies providing a comprehensive analysis on the impact of AS events from immune-related perspectives. Therefore, it is imperative to identify the potential regulatory relationships between AS events and prognosis and the immune microenvironment in HCC.

In our study, based on transcriptional data and clinical information of HCC patients downloaded from TCGA data portal, we utilized the ESTIMATE algorithm to calculate the immune and stromal scores of every HCC patient included and implemented Kaplan–Meier curves to explore prognostic differences between high/low immune/stromal score groups. We then identified the DEASs in HCC combined with the transcriptional data and evaluated them in functional enrichment analysis to explore the potential biological functions and signaling pathways of these events. Furthermore, a prognostic model was constructed based on the optimal DEASs identified by Cox regression analyses to verify their prognostic value. Finally, GSVA was performed to determine the complexity and multidimensional aspects of microenvironment formation and immune infiltration distribution in HCC, which may shed light on the current bottleneck facing HCC immunotherapy.

## Methods

### Data collection

Data for this study were derived from public databases. Transcriptional data and clinical information of HCC (hepatocellular carcinoma, C22.0) patients were downloaded from TCGA data portal (https://tcga-data.nci.nih.gov/tcga/). We employed the ESTIMATE algorithm in R software to calculate immune and stromal scores for the mRNA expression data (Yoshihara et al. 2013), a method that uses gene expression signatures to infer the fraction of stromal and immune cells in tumor samples, with the purpose of elucidating the promoting effect of the microenvironment on tumor cells, providing new thinking on the context of the evolution of genomic changes. Specifically, ESTIMATE outputs stromal and immune scores by performing ssGSEA. For a given sample, gene expression values were rank-normalized and rank-ordered. The empirical cumulative distribution functions of the genes in the signature and the remaining genes were calculated. A statistic was calculated by integrating the difference between the empirical cumulative distribution function, which is similar to the one used in gene set-enrichment analysis but based on absolute expression rather than differential expression. In our research, the gene expression profile was downloaded from TCGA database and input into R software. Then, by implementing the ESTIMATE algorithm, the immune score was automatically output. Similarly, stromal scores were also obtained by performing the above method (Yoshihara et al. 2013).

Finally, 370 HCC patients with complete transcriptional data and corresponding clinical information were selected for further analyses. In addition, we downloaded AS event data from TCGA SpliceSeq database for subsequent research. There has been wide consensus that the goal of using Percent Spliced In (PSI) (Ryan et al. 2012), ranging from 0–1, is to quantify events. Then, a strict set of screening conditions (sample percentage with PSI value of 75 and average PSI value of 0.05) was set to ensure the reliability of AS events included in subsequent analyses.

### Calculation and prognostic significance of stromal and immune scores

Stromal and immune scores were calculated by applying the ESTIMATE algorithm, and the R script was downloaded from the website (https://sourceforge.net/projects/estimateproject/). In addition, the X-tile program, a bioinformatics tool, was used to estimate the optimal cutoff values of big data analytics (Camp et al. 2004). Based on the results of ESTIMATE analysis, corresponding patients were classified into high/low immune score groups and high/low stromal score groups according to X-tile software. Then, the prognostic significance for each group was examined by applying Kaplan–Meier survival curves.

### Screening of differentially expressed AS events (DEASs) based on stromal and immune scores

To determine the reasons for the difference in prognosis between the high and low immune score groups, as well as the high and low stromal score groups, differential expression analyses of the PSI values of AS events were conducted. Given that the PSI values of many AS events were relatively small, we set a restricted condition of | logfc |> 0 and FDR/adjusted P < 0.05 to represent the upregulation and downregulation of relevant AS events, respectively, as previously described (Huang et al. 2019). Heatmaps and volcano plots were generated using the pheatmap package and ggplot2 in R software, respectively. As a result, intersecting AS events that were upregulated or downregulated in both the high immune and stromal score groups were selected as DEASs and screened out for further analysis using a Venn plot. Moreover, the UpSet plot was generated by the UpsetR package in R to visualize the intersections between the seven types of differentially expressed AS events in hepatocellular carcinoma (Conway et al. 2017).

### Functional enrichment analysis

Based on the DEASs above, functional enrichment analysis was employed for the Gene Ontology (GO) terms and the Kyoto Encyclopedia of Genes and Genomes (KEGG) pathways to assess the functional role of intersecting genes in Metascape (www.metascape.org). A P-value < 0.05 was set as the cutoff to identify significant events. The first 20 important terms in the GO analysis and the first 16 significant pathways in the KEGG are displayed in the bar charts.

### Construction of the prognostic model based on DEASs

With the aim of illustrating the prognostic value of DEASs in HCC patients, univariate Cox regression analysis was applied to identify survival-related DEASs. We then employed least absolute shrinkage and selection operator (LASSO) regression to identify the final elimination of potential predictors with nonzero coefficients (Gao et al. 2010), which avoids model overfitting to obtain the simplest (smallest parameter) model. Furthermore, multivariate Cox regression analysis was adopted to comprehensively evaluate the contribution of each DEAS to prognosis based on the statistics of negative log-likelihood and Akaike Information Criterion (AIC), confirming DEASs involved in the final prognostic signature. According to the results of the multivariate Cox regression analysis and the PSI values, we calculated the risk scores of HCC patients. The risk score was obtained by the following formula: $score =={\sum }_{i=0}^{n}\mathrm{PSI}\times {\beta }_{i}$, where β is the regression coefficient. HCC patients were divided into low- and high-risk groups based on their median risk score, and Kaplan–Meier survival curves were plotted to show the different prognoses. Additionally, receiver operating characteristic (ROC) curves of 1, 3, and 5 years were generated to display the discrimination of the predictive signatures.

### Independence of the risk score prognostic mode

HCC patients with full clinical parameters, including sex, age, TNM stage, American Joint Committee on Cancer (AJCC) stage and histologic grade, were subjected to analyses to validate the independence of the risk score based on survival-associated DEASs. We then conducted univariate and multivariate Cox regression analyses.

### Gene set variation analysis (GSVA) between high- and low-risk groups

GSVA, a gene set enrichment method that estimates variation of pathway activity over a sample population in a nonparametric and unsupervised manner, showed an increased ability to deal with molecular profiling experiments compared to other methods (Sonja Hänzelmann and Guinney 2013). Therefore, GSVA enrichment analysis is a robust choice to further mine differences in the activation status of biological pathways between the high- and low-risk groups, which was performed using the “GSVA” R package. The gene sets of “c2.cp.kegg.v7.1.-symbols” were downloaded from the MSigDB database for GSVA. An adjusted P-value < 0.05 was considered statistically significant.

### Statistical analysis

All statistical analyses were conducted in R software 3.6.1. All statistical tests with p < 0.05 (two-sided) were considered statistically significant.

## Results

### Association of immune and stromal scores with HCC prognosis

**Fig. 1:**
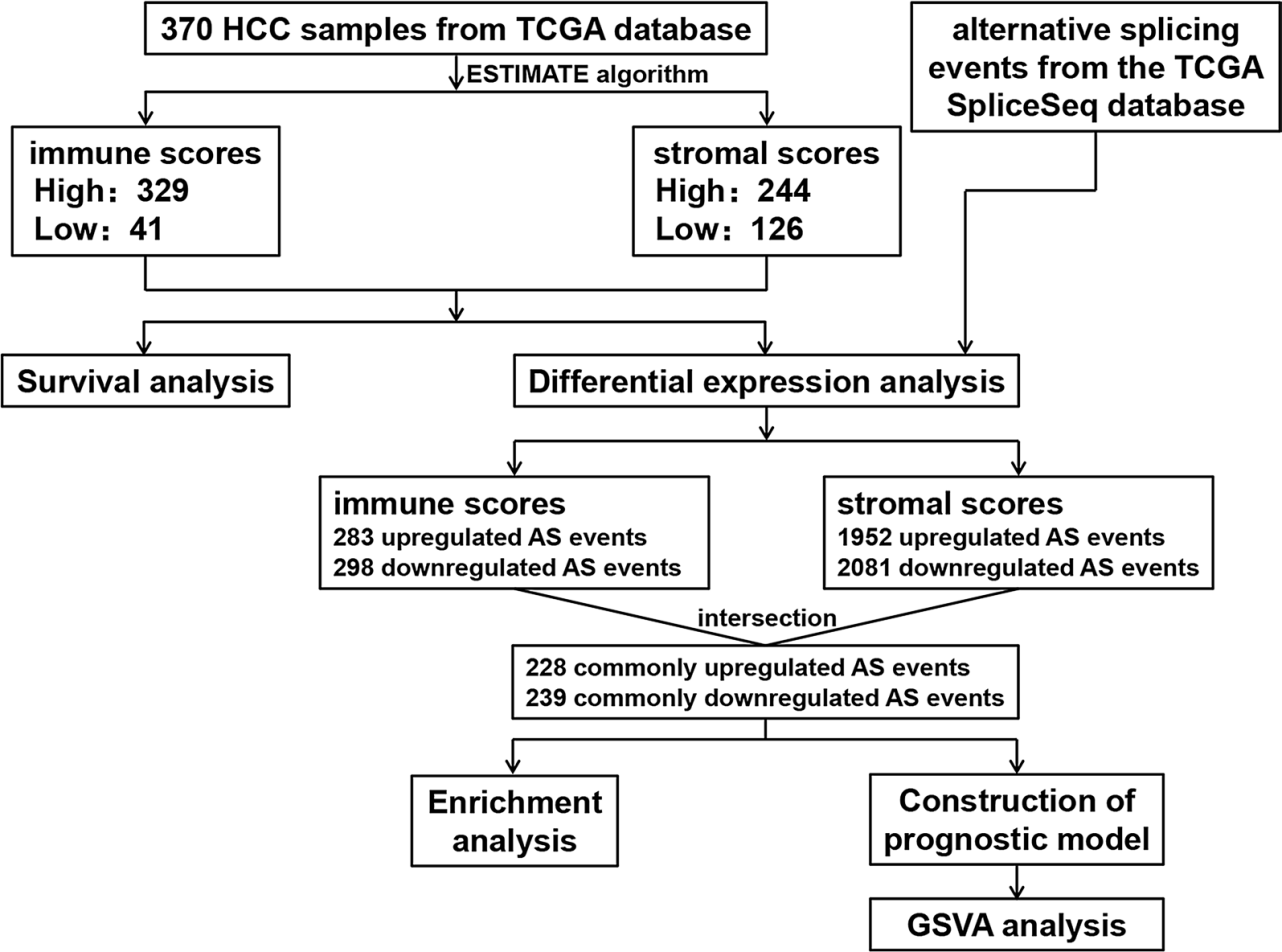
The workflow of the study. *HCC* hepatocellular carcinoma, *TCGA* The Cancer Genome Atlas database, *AS* alternative splicing

**Fig. 2:**
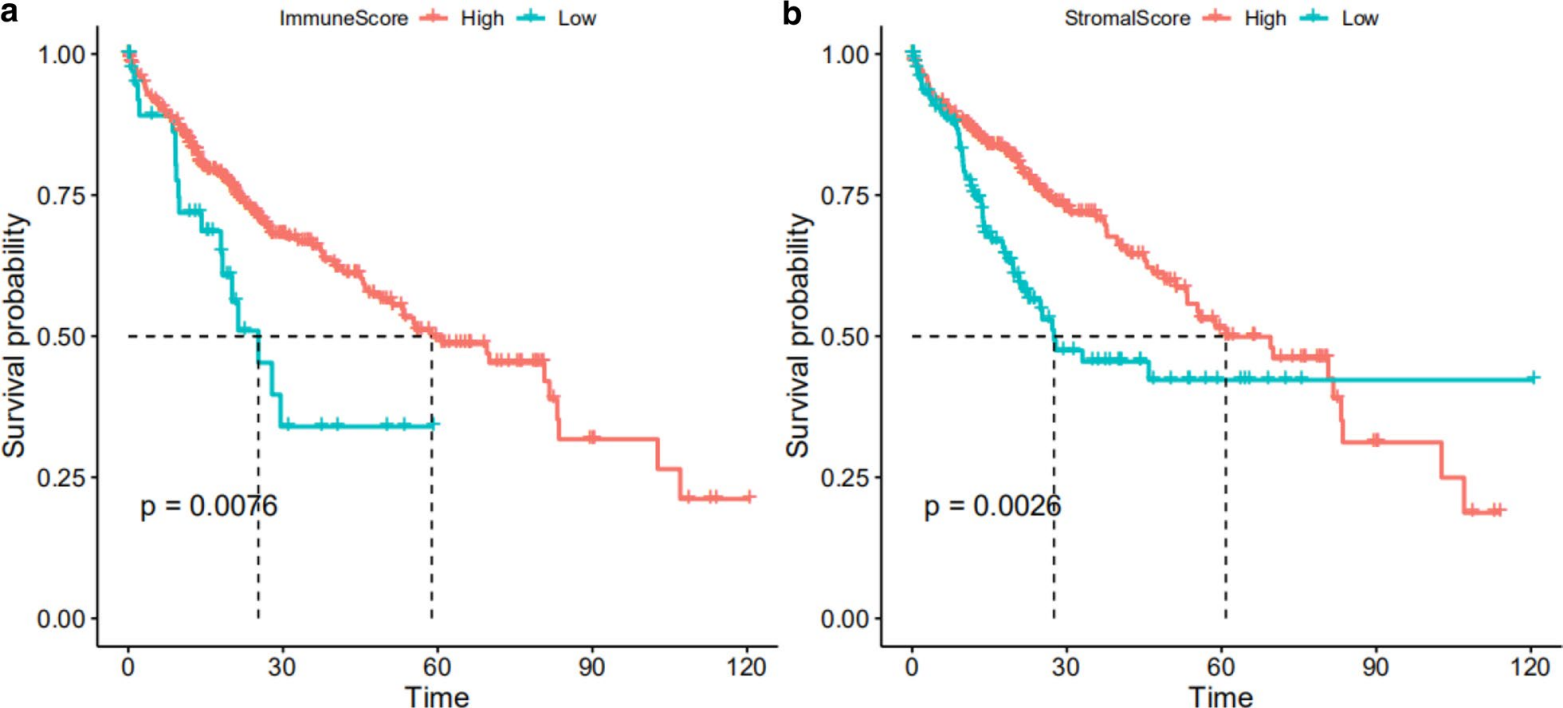
Association of stromal and immune scores with HCC patient prognosis. **a** Kaplan–Meier survival curves for patients with low and high immune scores. **b** Kaplan–Meier survival curves for patients with low and high stromal scores

The workflow of our study is shown as Fig. 1. A total of 370 HCC patients with complete clinical data and transcriptome data from TCGA database were included in the follow-up study, and their baseline characteristics are detailed in Table 1. According to ESTIMATE analysis, immune scores were distributed between − 861.77 and 3157.28, and stromal scores ranged from − 1622.33 to 1180.26. HCC patients were subsequently divided into high- and low-score groups according to the immune/stromal score to investigate their prognostic value. The cutoff value was determined using the X-Tile software. Kaplan–Meier survival curves showed that the high immune score group had a longer survival probability than the low immune score group (P = 0.0076); additionally, patients with a high stromal score had a longer survival probability (P = 0.0026) (Fig. 2a, b). These results indicate that immune and stromal scores are both significantly correlated with the prognosis of HCC patients and are worthy of further study.

**Table 1:** Patient characteristics at baseline

Characteristic	Number (%)
*Diagnosis age*	
< 60	201 (54.3)
≥ 60	169 (45.7)
Median age (range), years	53 (16–90)
*Gender*	
male	250 (67.6)
female	120 (32.4)
*Origin (race)*	
Asia	158 (42.7)
Non-Asia	212 (57.3)
*Ethnicity*	
Hispanic or Latino	132 (35.7)
Not Hispanic or Latino	227 (61.4)
NA	11 (2.9)
*ECOG performance status score*^*a*^	
0	162 (43.8)
1	84 (22.7)
2	26 (7.0)
> 2	98 (26.5)
*Child–Pugh classification grade*^*b*^	
A	216 (58.4)
B	21 (5.7)
C	1 (0.2)
NA	132 (35.7)
*Disease Stage (American Joint Committee on Cancer)*	
Stage I–II	256 (69.2)
Stage III	85 (23.0)
Stage IV	5 (1.3)
NA	24 (6.5)
*AFP*	
≥ 400 ng per milliliter	64 (17.3)
< 400 ng per milliliter	213 (57.6)
NA	93 (25.1)
*Family history of cancer*	
Yes	112 (30.3)
No	207 (55.9)
NA	51 (13.8)
*Histologic grade*^*c*^	
G1	55 (14.9)
G2	177 (47.8)
G3	121 (32.7)
G4	12 (3.3)
NA	5 (1.3)
*Adjacent hepatic tissue inflammation extent type*	
None	117 (31.6)
Mild	99 (26.8)
Severe	17 (4.6)
NA	137 (37.0)

^**a**^Eastern Cooperative Oncology Group (ECOG) scores range from 0 to 5, with higher numbers indicating poorer health

^b^The Child–Pugh classification grade is a three-category scale (A, with scores of 5 or 6, indicating good hepatic function; B, with scores of 7 to 9, indicating moderately impaired hepatic function; or C, with scores of 10 to 15, indicating advanced hepatic dysfunction). Classification is determined by scoring according to the presence and severity of five clinical measures of liver disease (encephalopathy, ascites, bilirubin levels, albumin levels, and prolonged prothrombin time)

^c^The histologic grade of hepatocellular carcinoma is based on Edmondson's classification, which can be divided into four grades. The higher the grade, the worse the differentiation

### Identification of DEASs in HCC

**Fig. 3:**
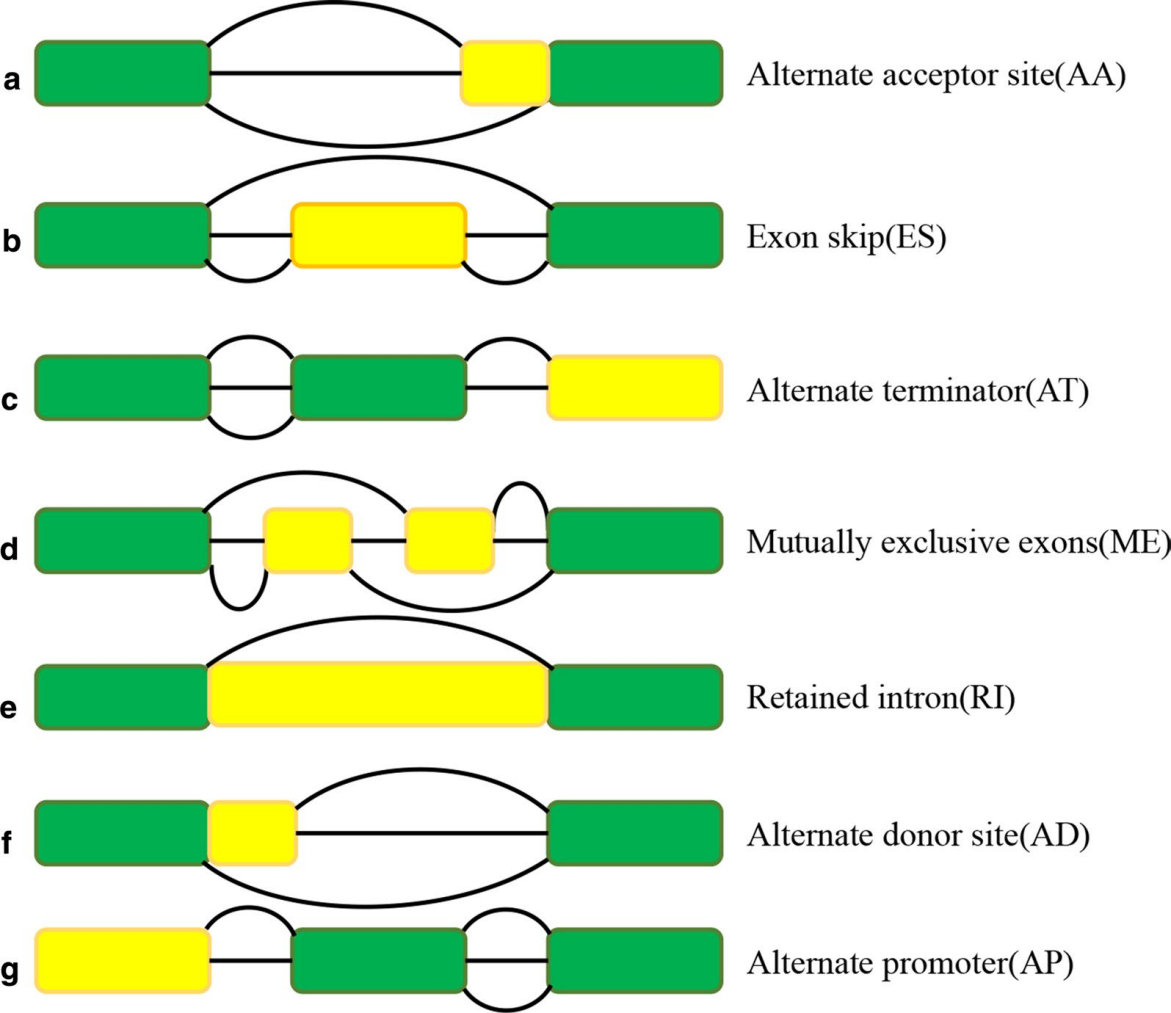
A schematic showing the seven types of alternative splicing examined in this study. **a** Alternate acceptor site, AA; **b** exon skip, ES; **c** alternate terminator, AT; **d** mutually exclusive exons, **e** ME; retained intron, RI; **f** alternate donor site, AD; **g** alternate promoter, AP

**Fig. 4:**
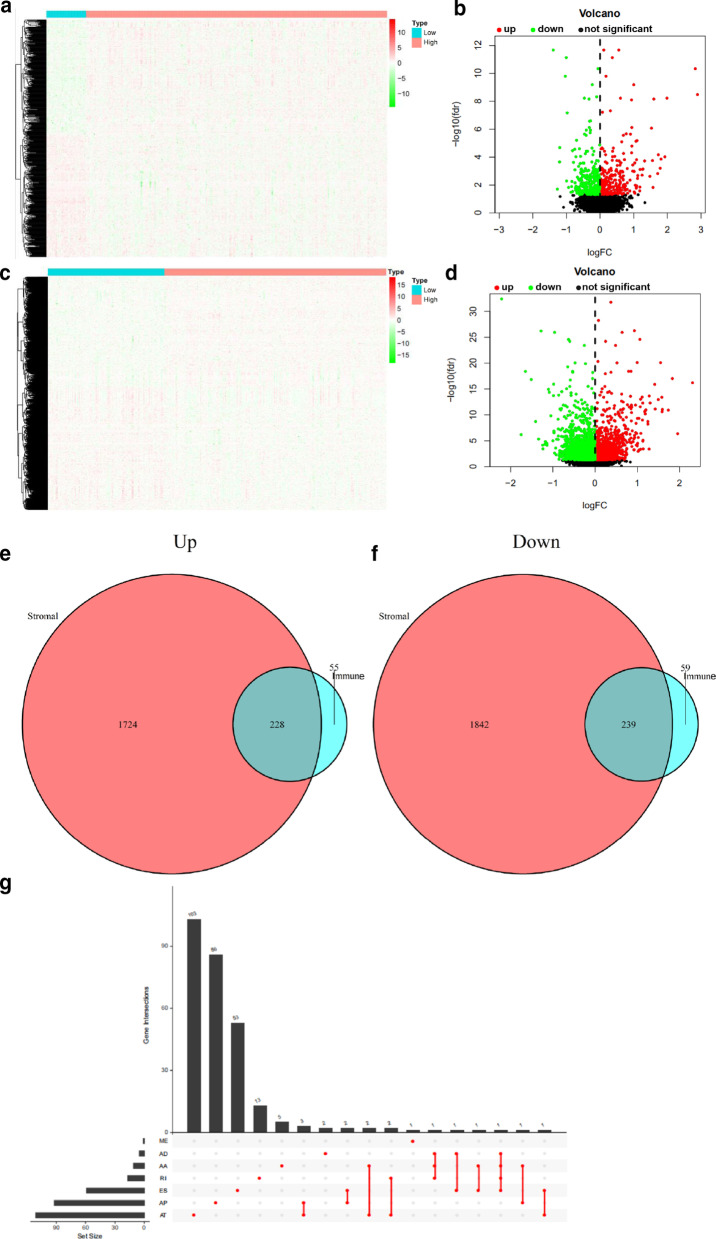
Identification of DEASs in HCC based on immune and stromal scores. **a**, **c** Heatmaps of AS event expression in the high and low groups based on immune and stromal scores. Red indicates that AS events with higher expression levels, and green indicates AS events with lower expression in the high-score groups. **b**, **d** Volcano plots of AS event expression in the high and low groups based on immune and stromal scores. The red and green points in the plots represent upregulated and downregulated AS events, respectively. **e**, **f** Venn diagram analysis of aberrantly expressed AS events between the immune and stromal score groups. **e**) Commonly upregulated AS events. **f** Commonly downregulated AS events. **g** UpSet plot of interactions among the seven types of DEASs

Increasing evidence has revealed that AS events play an important role in the development of cancer and the formation of the tumor microenvironment (Li et al. 2019; Zhang et al. 2019). In addition, to establish a reasonable relationship between immune/stromal scores and transcriptional data in the hope of finding other explanations for its influence on the prognosis of HCC patients, we downloaded data regarding AS events from TCGA SpliceSeq database, of which there are seven types (alternate acceptor site, AA; exon skip, ES; alternate terminator, AT; mutually exclusive exons, ME; retained intron, RI; alternate donor site, AD; and alternate promoter, AP). A schematic diagram illustrating the seven types is shown in Fig. 3. Then, we conducted differential analysis of AS event expression in the high and low groups based on immune and stromal scores, as reflected in the heatmaps (Fig. 4a, c). According to the analysis of immune scores, 283 upregulated and 298 downregulated AS events were identified in the high immune score group (Fig. 4b). Similarly, 1952 upregulated and 2081 downregulated AS events were compared between the high and low stromal score groups (Fig. 4d). Among them, we believed that intersecting AS events were most likely to be associated with the prognosis of HCC patients. Therefore, 228 commonly upregulated AS events and 239 commonly downregulated AS events between the immune and stromal score groups were selected as DEASs, as demonstrated in the Venn diagrams (Fig. 4e, f. Considering that a gene may have more than one type of AS event, we performed an upset plot to show the distribution characteristics of AS events and their mutual intersections in multiple dimensions (Fig. 4g). Notably, AP and AT events were the highest in number among them. Most of the genes had only one type of AS, while a few have two or more than four. The different combinations of these AS events may provide the most reasonable explanation for the enrichment of transcriptome diversity.

### Functional enrichment analysis

**Fig. 5:**
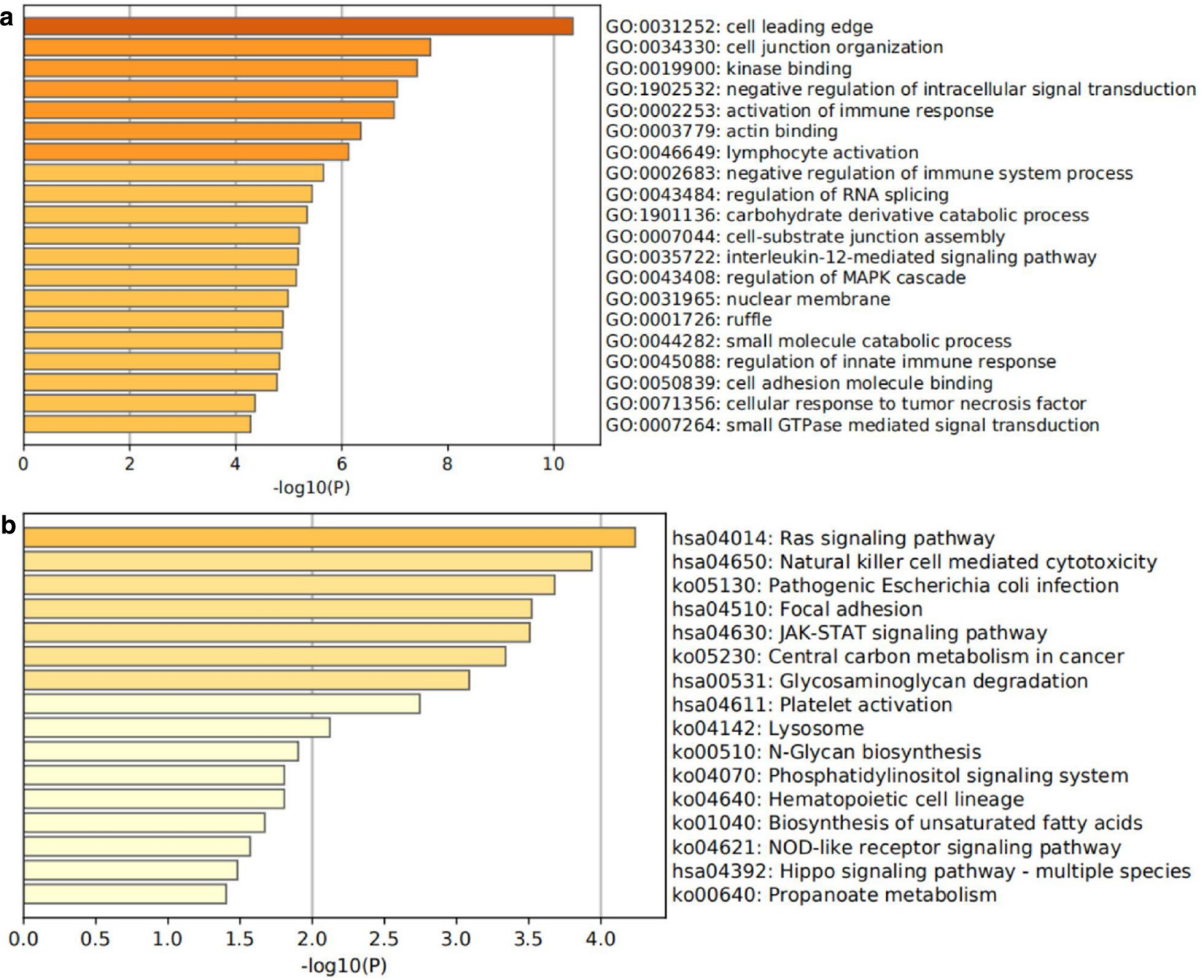
Enrichment analyses of the DEASs. **a** Bar graph of the top 20 results from the GO enrichment analysis. **b** Bar graph of the top 16 results from the KEGG enrichment analysis

To explore the potential biological functions and signaling pathways of DEASs, we conducted GO and KEGG pathway analysis. The top 20 results of GO analysis included “activation of immune response”, “lymphocyte activation”, “negative regulation of immune system process”, “interleukin-12-mediated signaling pathway”, “regulation of innate immune response”, and “cellular response to tumor necrosis factor” (all P < 0.05), in which immune-related responses accounted for the majority of results (Fig. 5a). Intriguingly, we also observed that genes enriched in GO categories, such as “negative regulation of intracellular signal transduction”, “regulation of RNA splicing”, “carbohydrate derivative catabolic process”, “regulation of MAPK cascade”, “small molecule catabolic process”, “cell adhesion molecule binding” and “small GTPase mediated signal transduction” (all P < 0.05), were closely related to HCC development. Consistent with these findings, KEGG pathways also revealed that some immune-related pathways were enriched, such as “natural killer cell mediated cytotoxicity”, “JAK-STAT signaling pathway”, and “NOD-like receptor signaling pathway” (all P < 0.05) (Fig. 5b). Moreover, other pathways associated with HCC tumorigenesis were enriched, including “Ras signaling pathway”, “focal adhesion”, “central carbon metabolism in cancer”, “phosphatidylinositol signaling system” and “Hippo signaling pathway”. Collectively, these outcomes suggest that DEASs may play important roles not only in the formation and shape of the HCC immune microenvironment but also in HCC tumorigenesis.

### Exploration of the prognostic value of DEASs

**Fig. 6:**
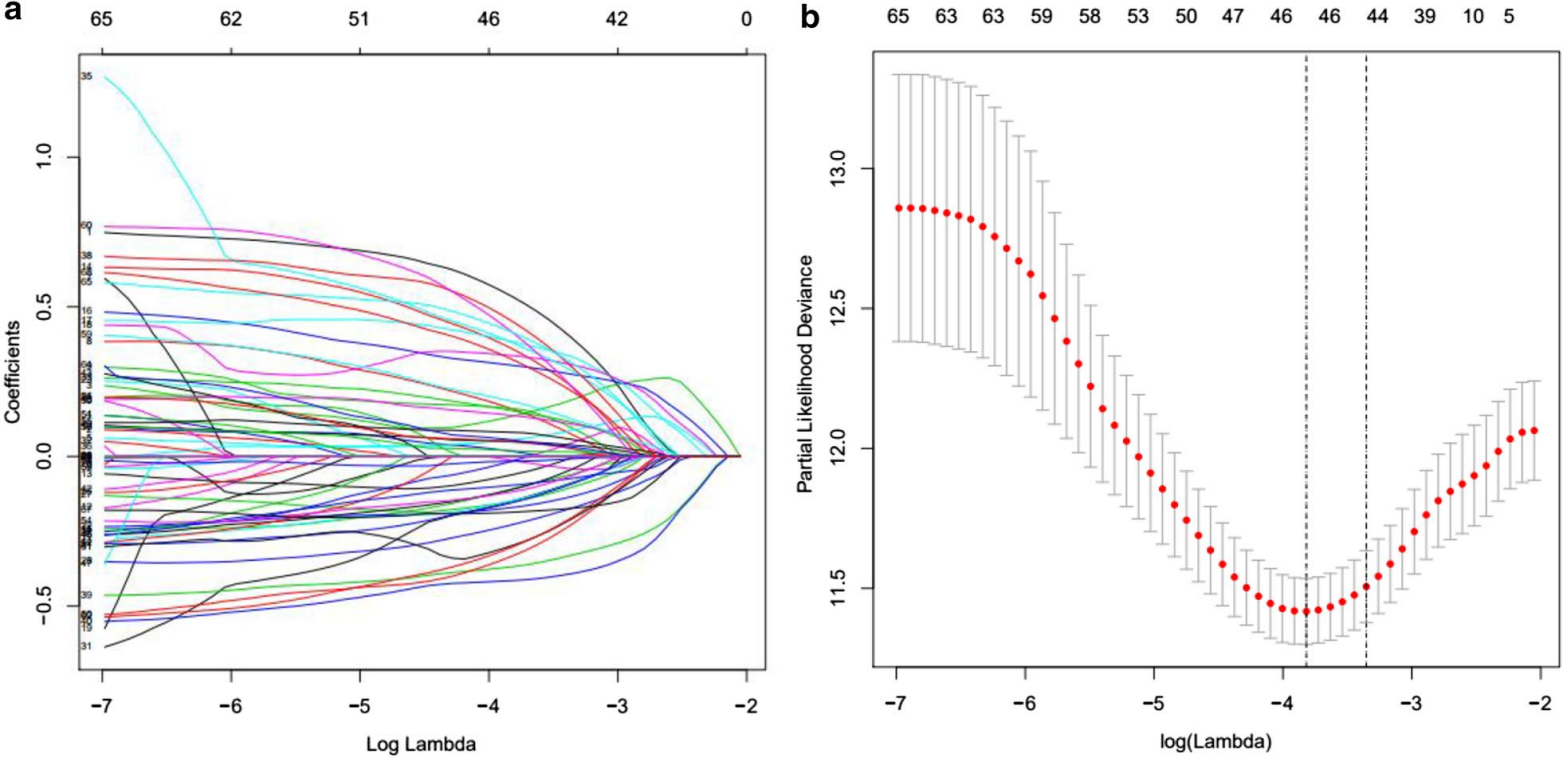
The optimal survival-related DEASs were selected for constructing the prediction model by LASSO regression. **a** LASSO coefficient profiles of the candidate survival-related DEASs. A coefficient profile plot was produced against the log λ sequence. **b** Dotted vertical lines were drawn at the optimal values using the minimum criteria.

**Fig. 7:**
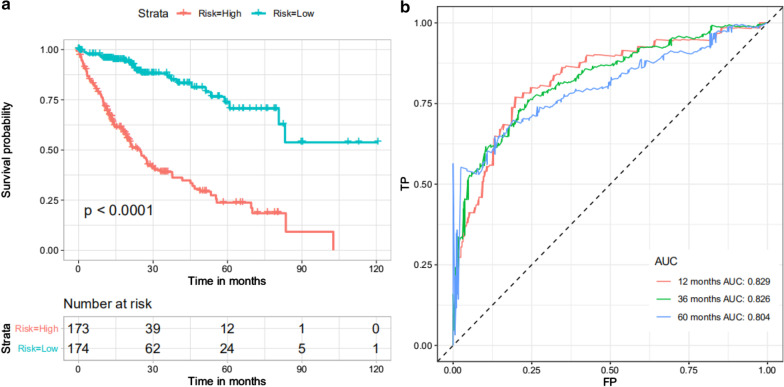
Exploration of the predictive ability of the prognostic model in the HCC cohort. **a** Survival analysis of the prognostic model. The upper panel shows the Kaplan–Meier curves for the high- and low-risk groups; the bottom panel shows the number of living patient variations with time in the high- and low-risk groups. Red represents the high-risk group, and blue represents the low-risk group. **b** ROC curves of predictive models at 1, 3, and 5 years. Red represents 1 year, green represents 3 years, and blue represents 5 years

Based on the DEASs, we constructed a signature to mine the underlying prognostic value of individual DEASs. First, we performed univariate Cox regression analysis. The results showed that 69 of the 467 intersecting AS events were significantly correlated with survival in HCC patients (Additional file 1). Then, LASSO regression was adopted to select the optimal survival-related DEASs to construct the prediction model to avoid model overfitting (Fig. 6a, b). Eventually, 16 DEASs were identified and included in the final prognostic signature by multivariate analysis (Table 2). In addition, the risk scores of each HCC patient were calculated according to the formula and the results of multivariate analysis, and all patients were divided into low- and high-risk groups based on the median risk score. As the Kaplan–Meier survival analysis shows, there were significant survival differences between the low- and high-risk groups (Fig. 7a). In other words, the low-risk group had a longer survival probability than the high-risk group (Fig. 7a). More importantly, the AUCs of the ROC curve at 1, 3, and 5 years ranged from 0.804 to 0.829, which verified the powerful predictive ability of the prognostic model (Fig. 7b).

**Table 2:** Identification of specific differentially expressed AS events involved in final prognostic signature by multivariate analysis

id	coef.	HR	95% CI	P-value
*B9D1*|39715|RI	1.08	2.95	1.94–4.50	0.00
*MYL6*|22376|AT	0.42	1.52	0.98–2.36	0.06
*FGL1*|82824|AP	0.58	1.78	1.21–2.62	0.00
*TUBB3*|38167|AP	0.55	1.74	1.16–2.61	0.01
*NICN1*|64872|ES	− 0.48	0.62	0.42–0.91	0.01
*TNIP1*|74126|AP	0.71	2.04	1.37–3.04	0.00
*CALD1*|81858|AP	0.96	2.61	1.67–4.08	0.00
*ARPP19*|30674|ES	− 0.58	0.56	0.38–0.83	0.00
*ARHGEF1*|50101|ES	− 0.58	0.56	0.37–0.84	0.01
*MAP7D3*|90197|AT	− 0.34	0.71	0.48–1.05	0.09
*IMPA1*|84296|ES	− 0.43	0.65	0.43–0.98	0.04
*FCGRT*|50957|AP	0.82	2.26	1.45–3.52	0.00
*ANKDD1A*|31138|AT	− 0.55	0.58	0.40–0.85	0.01
*SMARCC2*|22392|ES	− 0.45	0.64	0.43–0.94	0.02
*ZBP1*|59940|AT	0.69	1.98	1.33–2.97	0.00
*FUS*|36247|ES	0.61	1.84	1.25–2.69	0.00

*AS* alternative splicing, *coef* coefficient, *HR* hazard ratio

### Validation of the risk score as an independent prognostic factor

**Fig. 8:**
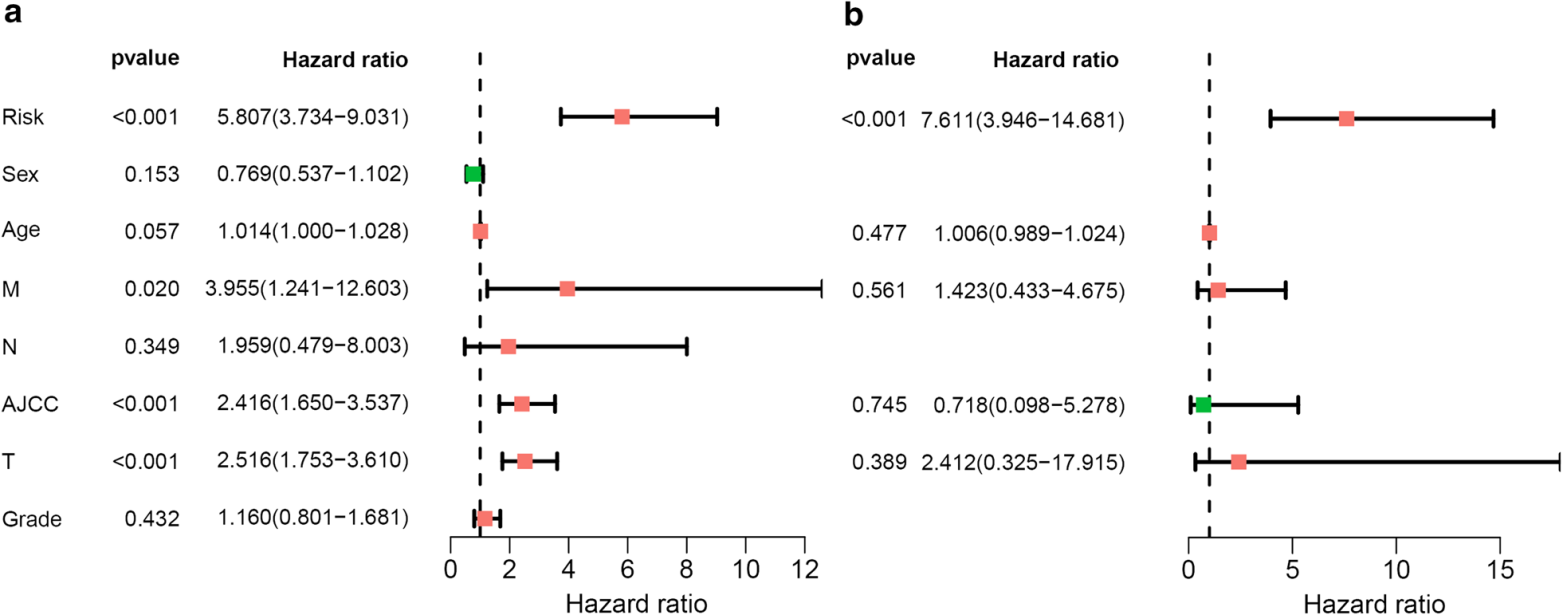
Verification of the independence of the risk score by Cox regression analyses. **a** Univariate Cox regression analyses incorporating the corresponding clinical information with the risk score. **b** Multivariate Cox regression analyses based on the clinical data and risk score

For a clearer understanding of whether the risk score was an independent prognostic factor in the stratified HCC cohorts, we performed univariate and multivariate Cox regression analyses involving risk score, sex, age, TNM stage, AJCC stage and histologic grade, which are shown in the form of forest plots (Fig. 8). Taken together, these results indicate that the risk score was an independent prognostic factor for HCC patient survival, irrespective of clinical parameters.

### Variation in immune-related pathways and biological process activity between high- and low-risk subgroups

**Fig. 9:**
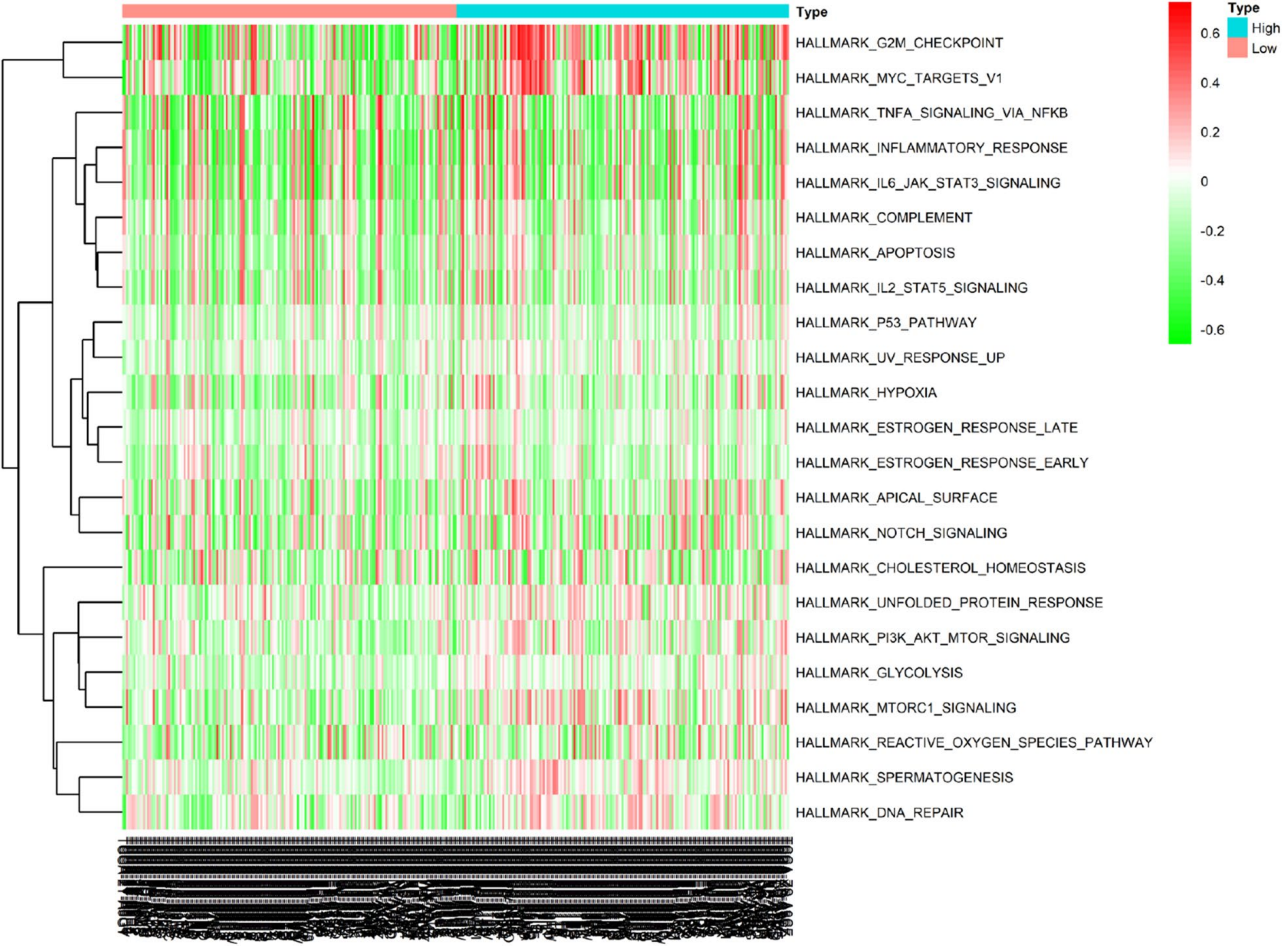
Differences in biological behaviors between the high- and low-risk subgroups evaluated by GSVA. The top 23 biological processes with significant differences are visualized by a heatmap. Red represents activated pathways, and green represents inhibited pathways

Driven by the outcomes of enrichment analysis and the prognostic model, we further employed GSVA to evaluate differences in biological behaviors between the high- and low-risk subgroups with the hope of obtaining a more comprehensive understanding of the prognostic differences (Fig. 9). As shown in Fig. 9, we noticed that the vast majority of enrichment pathways presented in the high-risk group were associated with carcinogenic activation pathways and processes, such as “glycolysis”, “mTORC1 signaling”, “hypoxia”, “PI3K-AKT-mTOR signaling”, “P53 pathway”, “NOTCH signaling”, “G2 M checkpoint”, “apoptosis”, “DNA repair” and “MYC targets V1”, or immune-related pathways, including “IL2-STAT5 signaling”, “inflammatory response” and “TNFa signaling via NF-kB”. These results reflect the complexity of the tumor microenvironment and multidimensional factors of tumor and immune microenvironment formation, which may lead to new insights into poor prognosis in high-risk groups.

## Discussion

In recent years, tumor immunotherapy, represented by immune checkpoint inhibitors, has made major breakthroughs, providing a new choice for the treatment of HCC (Kirkwood et al. 2012). However, current HCC immunotherapy still faces challenges, such as uncertainty in efficacy, numerous adverse events, and drug resistance even after initial patient benefits (Byun et al. 2017; Sharma et al. 2017). Previous studies have shown that the TME is closely related to the growth and development of HCC (Tahmasebi Birgani and Carloni 2017; Chen et al. 2020). Thanks to the tremendous development of bioinformatics and high-throughput technology, cancer genomics research has been greatly facilitated. Therefore, it is of cardinal significance to study the potential relationship between the TME and prognosis of HCC at the molecular level to guide the choice of clinical immunotherapy and combination therapy.

In the present study, we implemented the ESTIMATE algorithm to calculate immune and stromal scores of HCC derived from TCGA database through the specific perspective of the microenvironment. To predict the prognosis of HCC, we then adopted Kaplan–Meier curves and found that the high immune/stromal score group had a longer survival probability. By comparing transcriptional expression profiles in 370 HCC patients with high versus low stromal/immune scores, we identified 467 stromal/immune score-related DEASs and further selected the 16 optimal DEASs related to survival by LASSO Cox regression. Furthermore, the final prognostic signature was established, showing powerful predictive ability. More specifically, the low-risk group had a longer survival probability than the high-risk group, with AUCs of 1, 3, and 5 years ranging from 0.804 to 0.829. Additionally, the risk score can serve as an independent factor for predicting HCC patient survival. Collectively, this signature has great potential for predicting the survival of HCC patients.

Some genes in our prognostic signature model have been clarified to be involved in the progression of various tumors, including *FGL1* (fibrinogen-like protein 1), *TUBB3* (tubulin beta 3), *TNIP1* (TNF-α-induced protein 3-interacting protein 1), *CALD1* (caldesmon 1), *ARPP19* (cAMP-regulated phosphoproteins 19), *FCGRT* (Fc fragment of IgG receptor and transporter), *ANKDD1A* (ankyrin repeat and death domain containing 1A) and *SMARCC2* (SWI/SNF related, matrix associated, actin dependent regulator of chromatin subfamily C member 2) (Wang et al. 2019; Kanojia et al. 2020; Yang et al. 2019b; Liu et al. 2018; Ye et al. 2020; Xue et al. 2020; Feng et al. 2018; Yuan et al. 2018). Among these survival-related genes, *FGL1* and *ANKDD1A* were intriguingly closely associated with the tumor microenvironment and immune infiltration. *FGL1*, a liver-secreted protein, is a major *LAG-3* (lymphocyte-activation gene 3) functional ligand independent of MHC-II and is regarded as a novel immune evasion mechanism (Wang et al. 2019). In other words, blocking the FGL1-LAG-3 interaction with monoclonal antibodies stimulates tumor immunity and promotes a killing effect on tumors (Wang et al. 2019). On the other hand, previous research has demonstrated that loss of *FGL1* induces EMT (epithelial-mesenchymal transition) and angiogenesis in *LKB1* mutant lung adenocarcinoma, creating a tumor microenvironment more suitable for tumor growth and development (Bie et al. 2019). Regarding *ANKDD1A*, previous reports have suggested that it is a functional tumor suppressor gene, especially in the hypoxic microenvironment, that can inhibit the growth of glioblastoma multiforme (GBM) by inhibiting the transcriptional activity of hypoxia-inducible factor 1α (HIF1α), reducing the half-life of HIF1α, reducing glucose secretion, and inhibiting the production of lactic acid (Feng et al. 2018). In general, it primarily inhibits tumor growth by improving hypoxia in the tumor microenvironment and inhibiting glycolysis. However, *ANKDD1A* is highly methylated in tumors, indicating that it may be useful as a potential epigenetic biomarker and possible therapeutic target (Feng et al. 2018; Zhang et al. 2011).

Moreover, functional enrichment, including GO and KEGG analyses, suggested that DEASs were primarily involved in immune features, such as “lymphocyte activation”, “regulation of innate immune response”, “negative regulation of immune system process”, and “activation of immune response”, which not only includes the overall mobilization of adaptive immunity and innate immunity but also encompasses the positive and negative regulation of immunity, reflecting the complexity and dynamics of the tumor immune microenvironment and the diversification of the mechanism of AS events in the tumor immune microenvironment. In addition, we observed that some pathways associated with HCC tumorigenesis were enriched, such as “regulation of MAPK cascade”, “Ras signaling pathway”, “central carbon metabolism in cancer” and “Hippo signaling pathway”. Previous research has demonstrated that the Hippo signaling pathway is essentially a growth inhibition pathway mediated by a kinase cascade (Dong 2007). In the case of multiple signaling stimuli in the microenvironment, an imbalance in the Hippo pathway can lead to uncontrolled cell growth and malignant transformation, leading to the formation of malignant tumors (Harvey et al. 2013). In addition, studies have also shown that the Hippo signaling pathway not only directly regulates immune cells through activities such as affecting the differentiation of CD4 + helper T cells but also plays a regulatory role in the tumor microenvironment, such as recruiting additional type II macrophages and MDSCs and upregulating expression of the PD-L1 protein in tumor cells (Bhandoola 2020; Wang 2015; Janse van Rensburg 2018).

Additionally, GSVA of the high- and low-risk groups also aroused our interest. The tumor microenvironment is primarily composed of tumor-infiltrating lymphocytes, myeloid-derived cells, fibroblasts and other cellular components, as well as noncellular components, such as inflammatory factors and chemokines (Wu and Dai 2017). HCC is a typical inflammatory-related malignant tumor whose microenvironment contains a large number of macrophages and a series of innate and adaptive immune cells, forming complex immune tolerance microsurroundings (Kurebayashi 2018; Nishida and Kudo 2017). Interestingly, we found that the carcinogenic activation pathways significantly enriched in the high-risk group included many well-known signaling pathways related to metabolism, such as “glycolysis”, “mTORC1 signaling”, “hypoxia”, and “PI3K-AKT-mTOR signaling”. It is well known that carcinogenic signal transduction and metabolic changes are often interlinked in cancer cells, which utilize metabolic reprogramming to create a microenvironment suitable for their own growth to ensure survival and proliferation in the microenvironment under conditions of nutrient scarcity and hypoxia (Pavlova Natalya and Thompson 2016). Take “glycolysis” for example. Excessive conversion of sugar to lactic acid by tumor glycolysis inevitably leads to increased acidity of the tumor microenvironment. Studies have shown that the accumulation of lactic acid can induce macrophages to develop an inflammatory protumor phenotype, accelerating tumor progression and invasion (Paolini 2020). In addition, studies have shown that, affected by the higher glucose consumption rate of tumor cells, the mTOR activity of tumor infiltrating lymphocytes, the activation of T cell nuclear factor signals, and the ability of glycolysis are reduced, which leads to impaired antitumor effects (Chang 2015; Ho 2015). Collectively, taking into account factors such as metabolism, immunity, and tumorigenesis, this may provide a more comprehensive explanation for the poor prognosis of patients in the high-risk group; more interestingly, some of these results coincide with the enrichment analysis of DEASs. However, given the complexity and heterogeneity of the tumor immune microenvironment, further in-depth research is still necessary.

From the perspective of the microenvironment, we successfully identified prognostic AS events related to tumorigenesis and the immune microenvironment combined with transcriptome data. More importantly, we further constructed the final prognostic signature related to the stromal/immune score, showing satisfactory predictive ability. In addition, we did not identify DEASs through differential analysis of normal tissues and tumor tissues; rather, we identified target AS events through differential analysis of high/low immune-score groups and high/low stromal-score groups compared to other data in the literature. However, there are a few limitations to be considered in this study. First, all data come from the public TCGA database, so the possibility of selection bias cannot be ruled out. Second, due to the limited data included in TCGA at this stage, we were unable to include other clinical variables, such as past HBV infection history, family history, history of alcohol abuse, history of chronic liver disease, and chemoresistance. Therefore, based on the currently available data, we preliminarily conclude that risk is an independent prognostic factor. Of course, further research is needed to confirm these findings. In addition, we cannot conduct further independent database verification of the prognostic model due to a lack of relevant transcriptome data in other databases. Finally, our study is based on pure bioinformatics analysis and lacks relevant experimental validation at the basic or clinical level.

In summary, our research established a risk score model based on 16 prognostic DEASs to predict survival in HCC, which may help advance decision-making for personalized precision treatment. Notably, our study also elucidated the complexity and diversity of the immune microenvironment of HCC from an immunological point of view, providing one possible explanation for the lack of clinical efficacy observed in HCC patients.

## Conclusions

Taken together, the final prognostic signature formed with DEASs exhibited powerful prognostic value for predicting HCC outcomes. Even more thought-provoking is that there may be a vicious circle between the microenvironment characteristics of tumor tissue and tumor progression and patient survival. These results provide a new perspective for the implementation of clinical decision making and the development and optimization of immunotherapy for HCC patients, which provides a significant positive reference.

## Supplementary Information

**Additional file 1. **Screening differentially expressed AS events related to prognosis by univariate analysis in the HCC cohort.

## Data Availability

The datasets generated and/or analysed during the current study are available from the corresponding author on reasonable request.

## Abbreviations

HCC: Hepatocellular carcinoma
AS: Alternative splicing
DEASs: The differentially expressed AS events
GSVA: Gene set variation analysis
TCGA: The Cancer Genome Atlas
AA: Alternate acceptor site
ES: Exon skip
AT: Alternate terminator
ME: Mutually exclusive exons
RI: Retained intron
AD: Alternate donor site
AP: Alternate promoter
PSI: Percent spliced in
GO: Gene ontology
KEGG: Kyoto Encyclopedia of Genes and Genomes
LASSO: Least absolute shrinkage and selection operator
AIC: Akaike information criterion
ROC: Receiver operating characteristic
AJCC: American Joint Committee on Cancer
*FGL1*: Fibrinogen-like protein 1
*TUBB3*: Tubulin beta 3
*TNIP1*: TNF-α-induced protein 3-interacting protein 1
*CALD1*: Caldesmon 1
*ARPP19*: CAMP-regulated phosphoproteins 19
FCGRT: Fc fragment of IgG receptor and transporter
*ANKDD1A*: Ankyrin repeat and death domain containing 1A
*SMARCC2*: SWI/SNF related, matrix associated, actin dependent regulator of chromatin subfamily C member 2
*LAG-3*: Lymphocyte-activation gene 3
EMT: Pithelial-mesenchymal transition
GBM: Glioblastoma multiforme
HIF1α: Hypoxia-inducible factor 1α
